# Mechanism-based design of labile precursors for chromium(I) chemistry†

**DOI:** 10.1039/c5cc05993c

**Published:** 2015-10-28

**Authors:** Eser S. Akturk, Glenn P. A. Yap, Klaus H. Theopold

**Affiliations:** Department of Chemistry and Biochemistry, University of Delaware, Newark, DE 19716, USA

## Abstract

Dinitrogen complexes of the type Tp^R,R^Cr–N_2_–CrTp^R,R^ are not the most labile precursors for Cr(_I_) chemistry, as they are sterically protected from obligatory associative ligand substitution. A mononuclear alkyne complex – Tp^*t*Bu,Me^Cr(η^2^-C_2_(SiMe_3_)_2_) – proved to be much more reactive.

Half a century after the discovery of the first dinitrogen complex, by Allen and Senoff,^1^ coordination compounds of the rather inert N_2_ molecule are still much sought after, due in large part to their substitutional lability and concomitant role as precursors for a wide variety of transition metal complexes.^2^ For example, our interest in the activation of O_2_ and other small molecules has benefited greatly from the availability of Tp^*t*Bu,Me^Co(N_2_) and [(i-Pr_2_Ph)_2_nacnacCr]_2_(μ-η^2^:η^2^-N_2_), respectively.^3,4^ While these two molecules differ in the mode of coordination of the designated leaving group, both undergo facile ligand substitution to yield a plethora of compounds incorporating the Tp^*t*Bu,Me^Co and (i-Pr_2_Ph)_2_nacnacCr fragments.^5,6^ We were interested in the intersection of these two chemistries, and accordingly we now report the preparation of dinitrogen complexes of various TpCr fragments, which exhibited some notable differences in reactivity.

KC_8_ reduction under nitrogen of blue Tp^*t*Bu,Me^Cr(THF)Cl in Et_2_O/THF (4 : 1) at room temperature yielded green needles of [Tp^*t*Bu,Me^Cr]_2_(μ-η^1^:η^1^-N_2_) (**1**) in 42% yield (see ESI† for experimental detail and characterization of all compounds). The molecular structure of **1**, as determined by X-ray diffraction, is shown in Fig. 1. The dinuclear complex contains a single N_2_ ligand bridging the two staggered TpCr^I^ fragments, featuring end-on coordination of the dinitrogen to chromium. The N–N bond distance of 1.211(4) Å is substantially elongated over that of the free ligand (1.098 Å),^7^ and the Cr–N7 bond – at 1. 838(3) Å – is very short, certainly by comparison to the average Cr–N_Tp_ distance (2.198 Å). Both measures are consistent with strong π-backbonding from the low-valent chromium to the dinitrogen ligand. In accord with the crystallographically imposed inversion symmetry of **1**, its IR spectrum (KBr) did not show a discernable N–N stretching vibration. **1** is a paramagnetic substance with isotropically shifted and broadened ^1^H NMR resonances. At room temperature, it has an effective magnetic moment of *μ*_eff_ = 3.9(1) *μ*_B_, a possible interpretation of which is that the bridging N_2_ ligand mediates antiferromagnetic coupling between the two Cr^I^ (high-spin d^5^, *S* = 5/2) ions.

With **1** in hand, we embarked on an exploration of its reactivity with a variety of small molecules. As expected, the low-valent dinitrogen complex reacted rapidly with molecules that yielded products in which the chromium was oxidized. Examples include O_2_, S_8_, N_2_O, and RN_3_. While the chalcogenide chemistry will be detailed elsewhere, we offer the product of the reaction of 1 with adamantyl azide, *i.e*. purple Tp^*t*Bu,Me^Cr=NAd (**2**) as a representative example. **2** is the sole terminal imido complex of trivalent chromium.^8^ Its molecular structure is depicted in Fig. 2. The pseudo-tetrahedral complex features a linear imido ligand with a Cr–N distance of 1.687(2) Å; the latter is close the computationally predicted 1.708 Å for Tp^*t*Bu,Me^Cr=N^t^Bu.^9^ Consistent with the intermediate formal oxidation state of chromium it is also on the very long side of such distances.^10^ The effective magnetic moment of 2 measured *μ*_eff_ = 3.7(1) *μ*_B_, which is consistent with a quartet spin ground state (d^3^, *S* = 3/2).

To our surprise, reactions of **1** with good π-acceptors did either not proceed at all, or yielded decomposition products only after prolonged exposure. Thus, **1** did not react with alkenes (*e.g*., ethylene) or alkynes (*e.g*. 2-butyne), and lengthy exposure to an excess of CO (1 atm, 18 h) yielded only the ligand fragmentation product (^*t*Bu,Me^pzH)_2_Cr(CO)_4_, possibly due to traces of adventitious impurities (H_2_O?). We have reason to believe (*vide infra*) that all of these attempted reactions are thermodynamically favorable and would yield stable π-complexes. However, they apparently face insurmountable kinetic barriers, distinguishing **1** as a peculiarly substitution inert dinitrogen complex. To rationalize this disparity in reactivities, which has some precedent in titanium chemistry,^11^ we hypothesized that the reactions with oxidants may proceed via initial outer sphere electron transfer, thereby activating the Cr–N_2_ bond with respect to dissociation. Non-oxidizing ligands, on the other hand, may be forced to undergo an associative ligand substitution, because the Cr–N_2_ bond of **1** is too strong to permit a dissociative reaction path. The 13-electron configuration of the individual Cr atoms may make a ligand dissociation – yielding a bare, trigonal pyramidal 11-electron Tp^*t*Bu,Me^Cr fragment – energetically unfeasible. In this scenario, the effective steric shielding of the metal atoms by interleaving *tert*-butyl substituents of the opposing Tp^*t*Bu,Me^ ligands may prove impossible to penetrate, rendering the Cr–N_2_–Cr core of 1 impervious to ligand attack.

We then resolved to test the two essential pillars of this mechanistic hypothesis, namely (i) the lack of dissociation of **1**, and (ii) the steric blocking of associative ligand substitution pathways. A dissociation of **1** in the absence of N_2_ must yield either one or two equivalents of Tp^*t*Bu,Me^Cr or a solvate thereof (Tp^*t*Bu,Me^Cr(S), S = Et_2_O, THF). Alternatively, in the presence of gaseous N_2_, an associative reaction with the latter may produce two equivalents of mononuclear intermediate Tp^*t*Bu,Me^Cr(N_2_). Either way, the reversible dissociation into mononuclear fragments should lead to scrambling of mixtures of suitably labeled dinuclear N_2_ complexes. In order to test this prediction we have prepared [Tp^*t*Bu,iPr^Cr]_2_(μ-N_2_) (3), a close analog of 1. 3 has been fully characterized, and selected structural parameters are listed in Table 1. In a control experiment, the reduction of an equimolar mixture of Tp^*t*Bu,Me^Cr(THF)Cl and Tp^*t*Bu,iPr^Cr(^*t*Bu,iPr^pzH)Cl yielded a 1: 2: 1 mixture of **1**, [Tp^*t*Bu,Me^Cr](μ-N_2_)[CrTp^*t*Bu,iPr^], and **3**; the proportions of the products were measured by LIFDI-MS,^12^ which exhibited strong molecular ion (M^+^) peaks for these compounds. The ratio of the products did not change upon heating the mixture to reflux in THF. However, when a mixture of **1** and **3** in THF under vacuum was heated to 70 °C for two days, subsequent analysis of the mixture by LIFDI-MS showed no evidence for the formation of the mixed ligand complex ([Tp^*t*Bu,Me^Cr](μ-N_2_)[CrTp^*t*Bu,iPr^]). Similarly, when the same experiment was repeated under a N_2_ atmosphere, no signal for the mixed compound was detected in the mass spectrum. These results prove that **1** (and **3**) do not detectably dissociate in THF solution, even when heated for prolonged periods. A dissociative mechanism (I_d_ or D) for the ligand substitution of **1** is thereby ruled out.^13^

An alternative associative mechanism should be facilitated by lesser steric hindrance of the Tp ligands. To explore this possibility, we have prepared [Tp^iPr,iPr^Cr]_2_(μ-N_2_) (**4**). It is interesting to note that the N–N bond distance of **4** (see Table 1) does not significantly differ from those of **1** or **3**; the extent of π-backbonding is apparently similar in all three compounds. However, the Cr–N distances in **4** are appreciably shorter (by 0.066(2) Å), suggesting that lesser steric interactions between the opposing ligands allow for a closer approach of the two TpCr fragments. Space filling models of **1** and **4** (see Fig. S3, ESI†) also suggest greater accessibility of the chromium centers in **4**. In stark contrast to **1**, exposure of **4** to 1 atm of CO(g) resulted in an immediate color change from violet to yellow and precipitation of octahedral Tp^iPr,iPr^Cr(CO)_3_ (**5**, see Fig. S4, ESI†). It appears that the diminished steric protection of Cr by the Tp^iPr,iPr^-ligand causes a dramatic increase in the rate of ligand substitution; this observation argues strongly in favor of an associative substitution mechanism (I_a_ or A).

The results described above suggest that the preparation of coordination compounds of the Tp^*t*Bu,Me^Cr^I^ fragment will require a precursor that is subject to facile associative ligand substitution; in all likelihood this will require a mononuclear structure to disrupt the molecular sheath protecting the Cr–N_2_–Cr core of **1**. Based on related nacnacCr chemistry, and inspired by Rosenthal *et al*.,^14^ we selected Tp^*t*Bu,Me^Cr(η^2^-C_2_(SiMe_3_)_2_) (**6**) as a likely candidate.^15^ KC_8_ reduction of Tp^*t*Bu,Me^Cr(THF)Cl in Et_2_O/THF under vacuum in the presence of bis(trimethylsilyl)acetylene yielded brown crystals of **6** in 75% yield. The molecular structure of **6** (depicted in Fig. 3) features a severely distorted coordination environment, in which the centroid of the alkyne’s triple bond is displaced from the B–Cr axis of the threefold symmetric TpCr fragment by 49°. This *cis*-divacant octahedral structure creates two symmetry equivalent openings for attack by external ligands. The relatively long Cr–C_alkyne_ distances (2.048(2) and 2.084(2) Å) and the comparatively modest structural reorganization of the coordinated alkyne – by comparison with other complexes of the type Tp^*t*Bu,Me^Cr(η^2^-C_2_R_2_) (R = Me, Ph; see ESI†) – herald a rather tenuous hold of Cr upon this sterically encumbered alkyne. In accord with this notion, ‘spring-loaded’ **6** proved much more reactive toward ligand substitution than **1**!

The reactions of **6** with various π-acceptors are summarized in Scheme 1; the molecular structures of the products – as determined by X-ray diffraction – are included in the ESI.† When carried out in ethereal solvents (THF, Et_2_O), these reactions were facile and proceeded in good yield. The carbonylation of **6** is notable in that it stopped short of the formation of Tp^*t*Bu,Me^Cr(CO)_3_ (i.e., the analog of **5**). The actual product, κ^2^-Tp^*t*Bu,Me^Cr(CO)_2_(μ-η^1^:η^1^-CO)(Et_2_O)CrTp^*t*Bu,Me^ (7) is best rationalized as the product of a disproportionation, resulting in a mixed-valent (Cr^0^Cr^II^) isocarbonyl complex. The divalent chromium – formally a cation – has apparently lost its affinity for additional π-acids. The dinuclear ethylene complex, [κ^2^-Tp^*t*Bu,Me^Cr]_2_-(μ-η^2^:η^2^-C_2_H_4_) (**8**), while a rare case of ethylene π-bonded to two metals,^16^ finds precedent in the analogous [(i-Pr_2_Ph)_2_nacnacCr]_2_-(μ-η^2^:η^2^-C_2_H_4_).^4^ Like the latter, it did not react further with ethylene, exhibiting no activity for catalytic oligomerization or polymerization of ethylene.^6a^ The irreversible reactions of **6** with less hindered alkynes were expected, being of interest mostly for the formation of pseudotetrahedral alkyne complexes **9** and **10**, as evidenced by ^1^H NMR. More surprising was the observation that **6** reacted with N_2_ (1 atm), forming **1** and free alkyne quantitatively! The spontaneous substitution of an alkyne ligand by N_2_ is rather unusual. It is a measure of the instability and lability of **6** and – if additional proof was needed – suggests that it is an excellent precursor for Tp^*t*Bu,Me^Cr^I^ chemistry.

We are now exploring the small molecule activation chemistry of TpCr(_I_) fragments, judiciously using the synthons described above. The results of these studies will be reported in due course.

## Supplementary Material

SI

## Figures and Tables

**Fig. 1:**
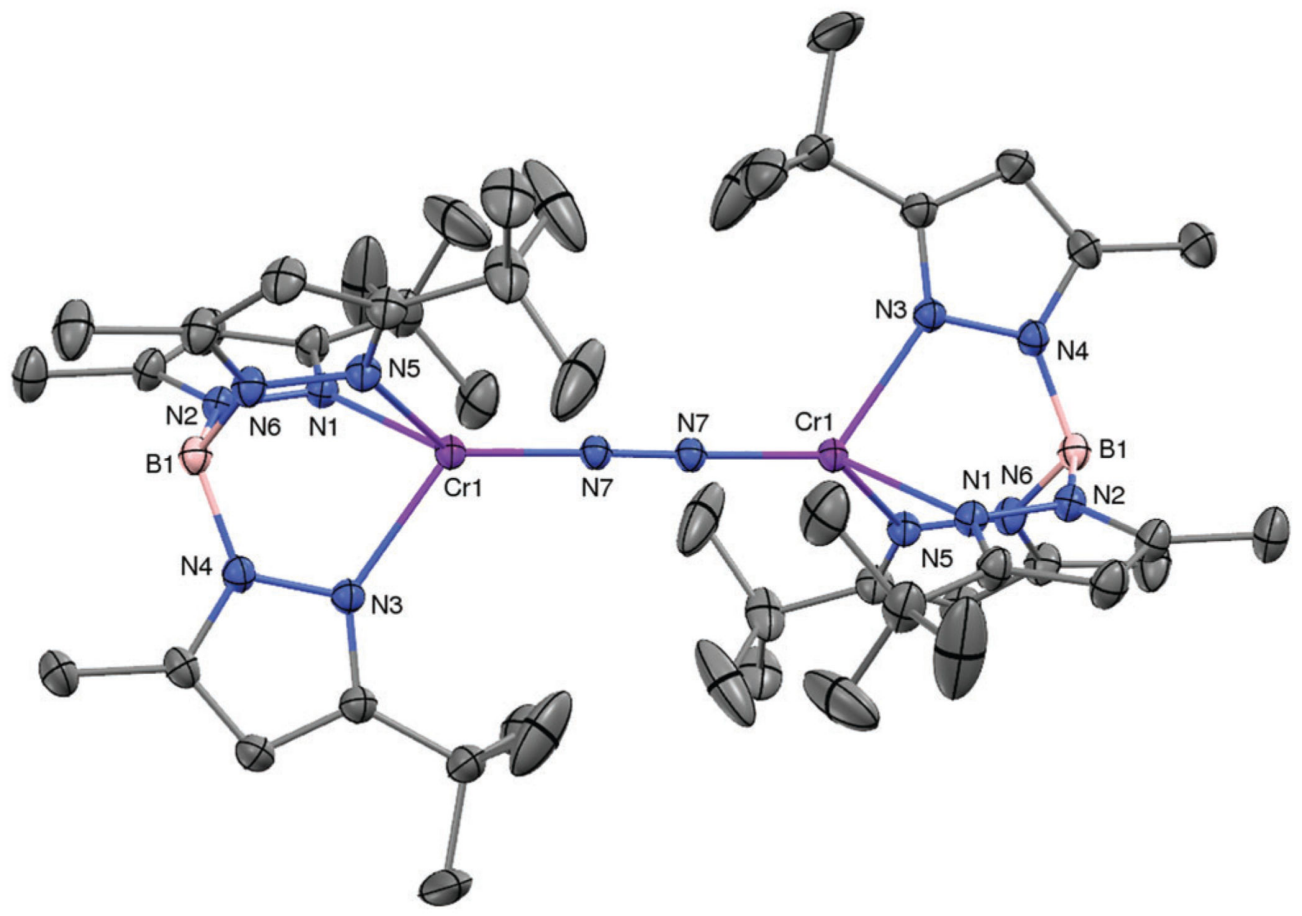
The molecular structure of [Tp^*t*Bu,Me^Cr]_2_(μ-η^1^:η^1^-N_2_) (**1**, 30% probability level). Selected interatomic distances (Å) and angles (°): N7–N7A, 1.213(5); Cr–N7, 1.838(3), Cr–N1, 2.205(3); Cr–N3, 2.200(3); Cr–N5, 2. 190(3); N_Tp_–Cr–N_Tp,avg_, 87.3; N_Tp_–Cr–N7_avg_, 127.2.

**Fig. 2:**
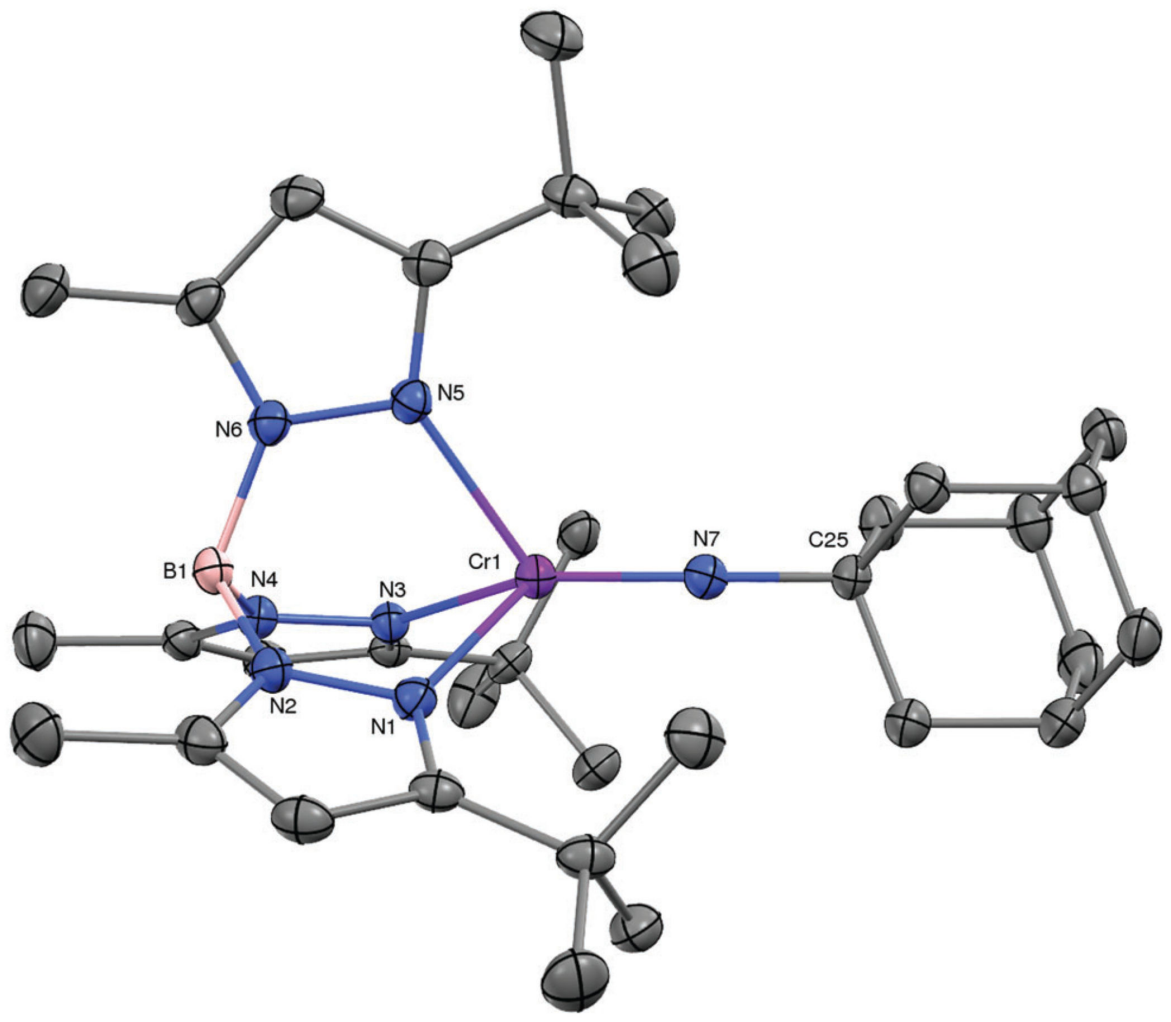
The molecular structure of Tp^*t*Bu,Me^Cr=NAd (**2**, 30% probability level). Selected interatomic distances (Å) and angles (°): Cr–N7, 1.687(2);N7–C25, 1.455(3); Cr–N1, 2.132(2); Cr–N3, 2.151(2); Cr–N5, 2.160(2); Cr1–N7–C25, 178.8(2)°; N_Tp_–Cr–N_Tp,avg_, 88.0; N_Tp_–Cr–N7_avg_, 126.7.

**Fig. 3:**
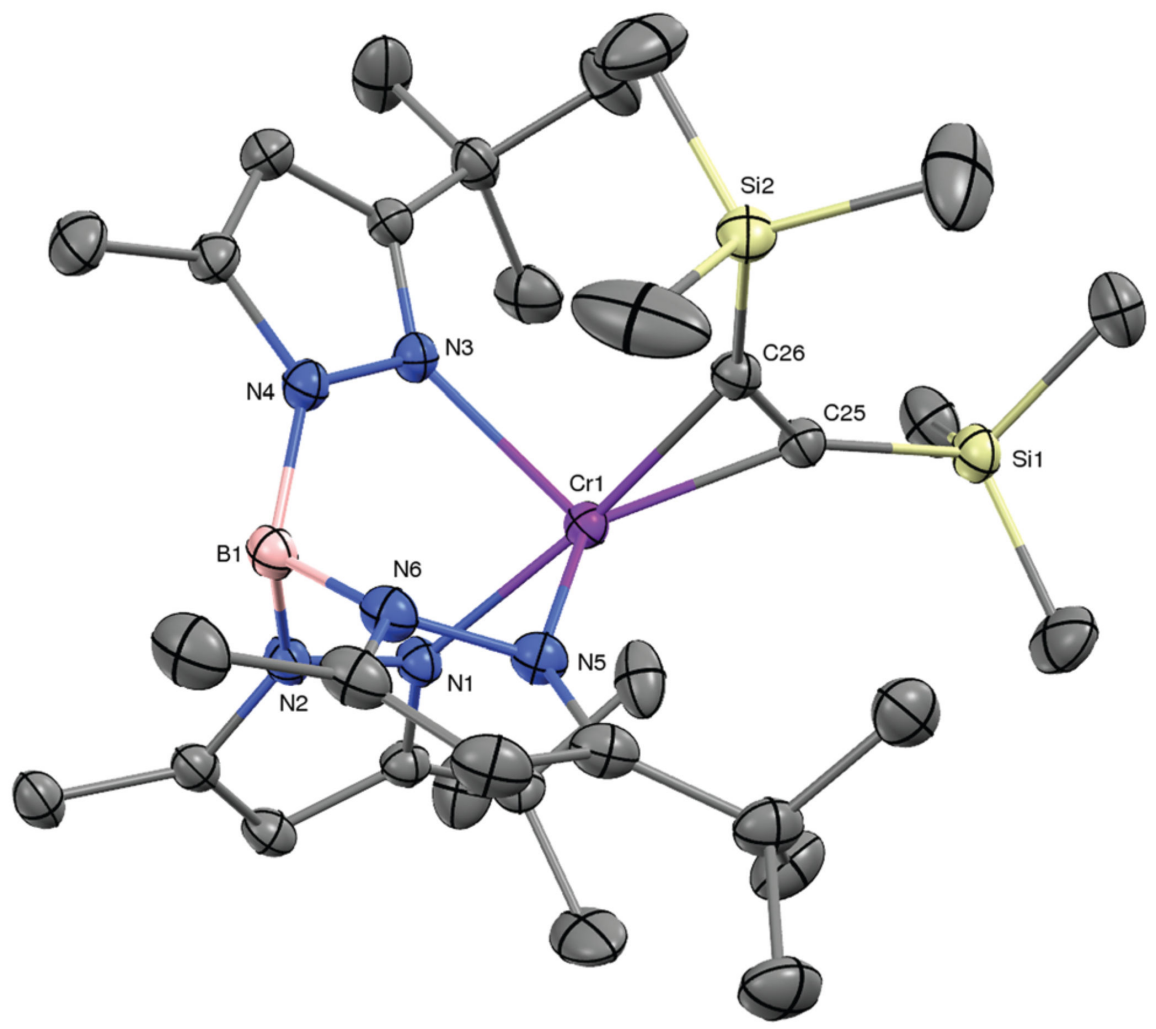
The molecular structure of Tp^*t*Bu,Me^Cr(η^2^-C_2_(SiMe_3_)_2_) (**6**, 30% probability level). Selected interatomic distances (Å) and angles (°): Cr–C25, 2.0480(19);Cr–C26, 2.0835(18); C25–C26, 1.288(3); Cr–N1, 2.1015(15); Cr–N3, 2.1614(16); Cr–N5, 2. 1504(16); N_Tp_–Cr–N_Tp,avg_, 87.7; N1–Cr–C25/C26_centroid_, 172.5; α (angle of deviation of alkyne centroid from B–Cr axis) = 49.3°.

**Scheme 1:**
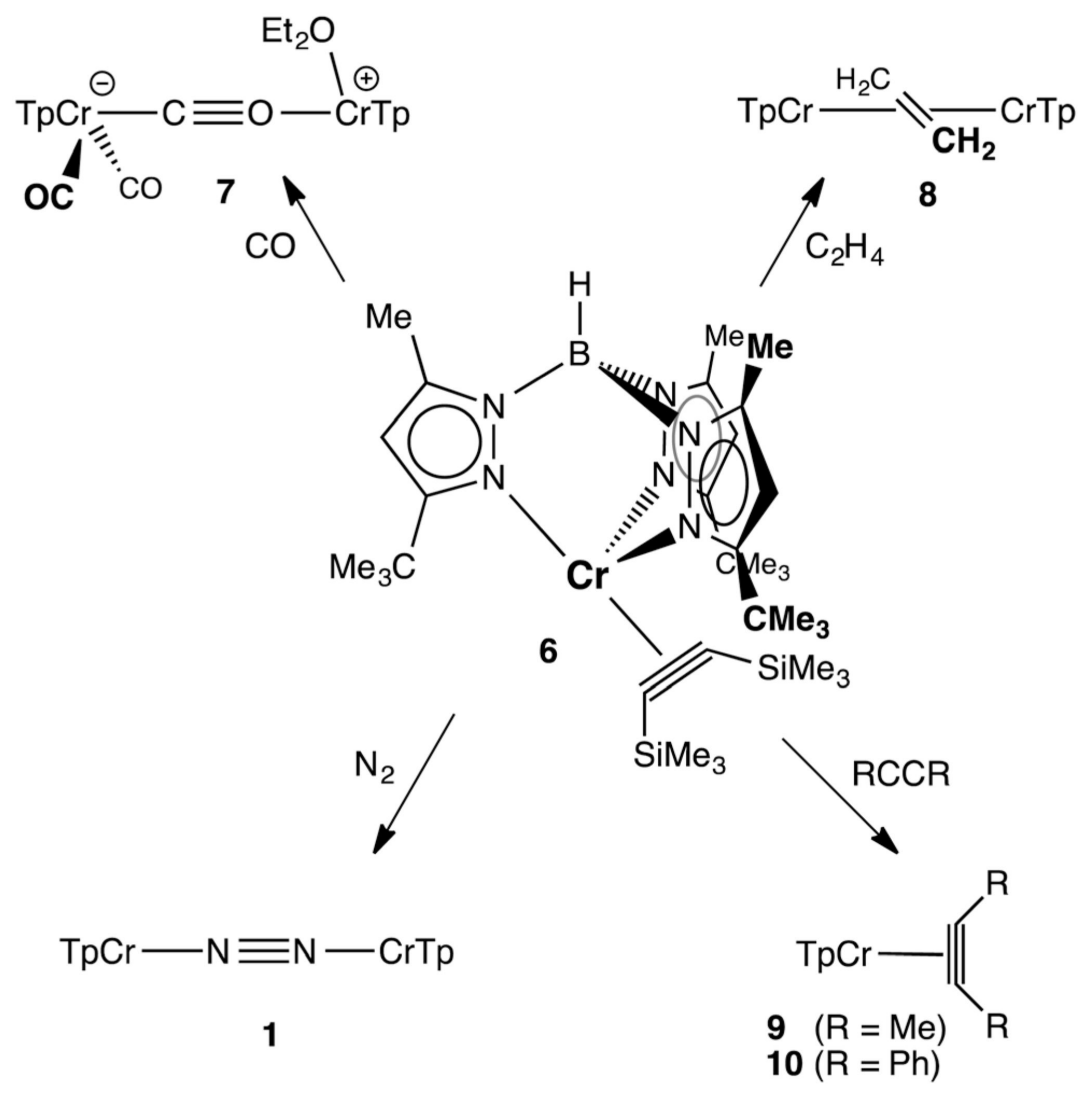
Ligand substitution reactions of **6**.

**Table 1:** Selected structural parameters of dinitrogen complexes [Tp^R,R′^Cr]_2_(μ-N_2_)

Compound	**1** (Tp^*t*Bu,Me^)	**3** (Tp^*t*Bu,iPr^)	**4** (Tp^iPr,iPr^)
N–N [Å]	1.213(5)	1.209(3)	1.214(4)
Cr–N [Å]	1.838(3)	1.8395(16)	1.773(2)
Cr–N_Tp_ [Å]	2. 198	2.191	2.094

## Footnotes

† Electronic supplementary information (ESI) available: Experimental details and molecular structures of **3–5** and **7–10**. CCDC 1058333–1058342. For ESI and crystallographic data in CIF or other electronic format see DOI: 10.1039/c5cc05993c
