# Supplementary material for: Mechanism-based design of labile precursors for chromium(I) chemistry
Source: Chem Commun (Camb). Author manuscript; Available in PMC 2016 Oct 28. (PMC4893307; doi:10.1039/c5cc05993c)
Supplement: SI [file NIHMS790264-supplement-SI.pdf]

Precursors for TpCr(I) chemistry – N_2 is not always a labile ligand.

Eser S. Akturk, Glenn P. A. Yap, and Klaus H. Theopold*

Department of Chemistry and Biochemistry, University of Delaware, Newark, DE 19716

Supporting Information

Contents:

- Synthesis and characterization of Cr complexes
- Single crystal X-ray structures

General:

All manipulations of compounds and reactions were performed in an inert atmosphere glovebox under an atmosphere of N_2 and glass ampoules using high vacuum line techniques. Solvents were purchased from Fisher Scientific and Thomas Scientific. All solvents were dried by passing through activated alumina and degassing prior to use. THF-d_8 and C_6D_6 were purchased from Cambridge Isotopes Laboratory and stored under vacuum over Na/K alloy. $Tp^{tBu,Me}CrCl$,[1] $[Tp^{iPr2}Cr]_2(\mu\text{-}Cl)_2$,[2] $TlTp^{tBu,Me}$, $TlTp^{iPr2}$, $TlTp^{tBu,iPr}$,[3] CrI_2,[4] and KC_8 [5] were synthesized according to literature procedures. Ethylene and carbon monoxide were purchased from Matheson. All other gaseous reagents were bought from Keen Compressed Gas Co. Organic reagents were purchased from Aldrich or Acros and used without further purification and manipulations while inorganic reagents were bought from Strem.

X-ray crystallographic studies were done in the X-ray crystallographic facility at the University of Delaware. ^1H NMR spectra were obtained from using a Bruker AVIII 400 spectrometer and were referenced to the residual solvent protons (C_6D_6 = 7.16 ppm, d_8-THF = 3.58 ppm and 1.73 ppm). FT-IR spectra were obtained using a Mattson Alpha Centauri or Nicolet Magna-IR 560 spectrometers (resolution = 4 cm^{-1}). LIFDI-MS were obtained using a Waters GCT Premier (High resolution time-of-flight mass spectrometer.

Elemental analyses were acquired from Robertson Microlit, Ledgewood, NJ 07852. A Johnson Matthey magnetic susceptibility balance was used to obtain room temperature molar magnetic susceptibilities (χ_m) in the solid state, which were corrected for diamagnetism using Pascal constants. These values were then converted into effective magnetic moments (μ_{eff}).

Preparation of [Tp$^{t\text{Bu,Me}}$Cr]$_2$(μ-N$_2$) (1)

Tp$^{t\text{Bu,Me}}$Cr(THF)Cl (1.00 g, 1.95 mmol) was added to an Et_2O/ THF mixture (v:v = 4:1). 1.2 equivalents of KC_8 (0.32 g, 2.35 mmol) was added gradually over the course of 2 minutes. The solution turned from blue to green by the end of the addition. The solution was allowed to stir for an additional four hours to ensure the complete reduction of Tp$^{t\text{Bu,Me}}$Cr(THF)Cl. The solvents were removed under vacuum. Toluene was added to the green residue and the solution was warmed to a gentle reflux. Graphite and KCl salt were then removed via filtration. The toluene solvent was removed under vacuum to yield a green residue. The solid was washed with pentanes, to remove any side products that can form from the over-reduction of Tp$^{t\text{Bu,Me}}$Cr(THF)Cl. The remaining green solid was collected. [Tp$^{t\text{Bu,Me}}$Cr]$_2$(μ-N$_2$) was recrystallized by cooling a saturated THF solution to -35° C overnight to yield green crystals (0.43 g, 42% yield). ^{1}H NMR (400 MHz, C_6D_6): 6.9 (b, 9H), 5.7 (br, 3H), 1.4 (br, 27H) ppm. IR (KBr): 2957 (s), 2928 (w), 2542 (w), 1542 (w), 1423 (w), 1358 (s), 1197 (s), 1066 (w), 783 (w), 649 (w) cm^{-1}. μ_{eff} (293 K) = 3.9(1) μ_B. M.p.: 252 – 254 °C. MS (LIFDI, toluene): *m/z* 978.5737 [M$^+$]; calcd: 978.5687. Anal. Calcd. for $C_{48}H_{80}N_{14}B_2Cr_2$: C, 58.90; H, 8.24: N, 20.03. Found: C, 56.56; H, 8.03; N, 16.28. Note: this complex is very air sensitive; the analytical results match $C_{24}H_{40}N_6BCrO_2$, i.e., the product of the reaction of [Tp$^{t\text{Bu,Me}}$Cr]$_2$(μ-N$_2$) with dioxygen. Anal. Calcd. for $C_{24}H_{40}N_6BCrO_2$: C, 56.79; H, 7.94: N, 16.55.

Preparation of Tp$^{t\text{Bu,Me}}$CrNAd (2)

[Tp$^{t\text{Bu,Me}}$Cr]$_2$(μ-N$_2$) (0.300 g, 0.307 mmol) was dissolved in 30 mL of THF. Two equivalents of adamantyl azide (0.108 g, 0.614 mmol) were added to the green solution. The color changed to purple over the course of 20 minutes of stirring. The solution was allowed to stir for an additional hour. The THF was removed yielding a purple residue,

which was recrystallized by cooling a saturated pentanes solution to -35 °C overnight, yielding purple crystals of **2** (0.372g, 95% yield). ^{1}H NMR (400 MHz, C_6D_6): 48.4 (br, 9H), 2.13 (br), 1.74 (br), 1.58 (br), 1.32 (br), 1.29 (s), 0.87 (s), -1.4 (br, 27H), -29.5 (br, 3H) ppm. IR (KBr): 2957 (s), 2904 (s), 2539 (w), 1541 (s), 1423 (s), 1356 (s), 1194 (s), 1066 (s), 788 (s), 722 (s), 648 (w) cm^{-1}. μ_{eff} (293K) = 3.7(1) μ_B. M.p.: 272 – 275 °C. MS (LIFDI, toluene): *m/z* 624.4025 [M^{+}]; calcd: 624.4017. Anal. Calcd. for $C_{34}H_{55}N_7BCr$: C, 65.37; H, 8.87: N, 15.70. Found: C, 62.45; H, 8.68; N, 14.59. Note: this complex is highly reactive and air sensitive. The analysis is more consistent with $C_{34}H_{55}N_7BCrO_2$, which may form when $C_{34}H_{55}N_7BCr$ reacts with O_2. Anal. Calcd. for $C_{34}H_{55}N_7BCrO_2$: C, 62.19; H, 8.44; N, 14.93.

Preparation of Tp^{tBu,iPr}CrCl(Pz^{tBu,iPr} H)

Cr(II)Cl_2 (0.0725 g, 0.589 mmol) was suspended in 50 mL of THF and allowed to stir for 5 minutes. TlTp^{tBu,iPr} (0.42 g, 0.589 mmol), in 50 mL of THF, was added slowly to the mixture over the course of 5 minutes. To this solution, Pz^{tBu,iPr}H (0.097 g, 0.589 mmol) was added . The solution turned blue and a white precipitate formed. The solution was allowed to stir for an additional 3 hours. The white solid was removed *via* filtration. The THF solvent was then removed under vacuum to yield a blue residue. The solid was redissolved into pentanes. Tp^{tBu,iPr}CrCl(Pz^{tBu,iPr}H) was recrystallized by cooling a saturated solution of the product in pentanes to -35° C overnight, yielding blue crystals (0.375g, 84% yield). ^{1}H NMR (400 MHz, THF-d_8): 25.2 (br), 15.5 (br), 9.6 (br), 4.2 – 3.7 (br), 1.8 (br), 1.3 – 0.2 (br), -24.7 (br) ppm. IR (KBr): 3283 (s), 3196 (s), 2965 (s), 2929 (s), 2867 (w), 2598 (w), 1559 (s), 1522 (s), 1465 (s), 1362 (s), 1270 (w), 1236 (s), 1171 (s), 1059 (s), 1014 (s), 799 (s), 644 (w) cm^{-1}. μ_{eff} (293 K) = 5.0(1) μ_B. M.p.: 245 – 246 °C. MS (LIFDI, toluene): *m/z* 760.4944 [M^{+}]; calcd: 760.4910. Anal. Calcd. for $C_{40}H_{70}N_8BCrCl$: C, 63.11; H, 9.27; N, 14.27. Found: C, 62.30; H, 9.24; N, 14.47.

Preparation of [Tp^{tBu,iPr}Cr]$_2$(μ-N_2) (3)

Tp^{tBu,iPr}CrCl(Pz^{tBu,iPr}H) (1.00 g, 1.32 mmol) was added to a mixture of Et_2O/THF (v:v = 4 : 1). KC_8 (0.213 g, 1.58 mmol) was added gradually over the course of 2 minutes, during which the solution turned from blue to green. The solution was allowed

to stir for an additional 6 hours to ensure complete reduction of $Tp^{tBu,iPr}CrCl(Pz^{tBu,iPr}H)$. The solvents were removed under vacuum. Toluene was added to the green residue and the solution was warmed to a gentle reflux. Graphite and KCl salt were then removed via filtration. The toluene solvent was removed under vacuum to yield a green residue. This solid was washed with pentanes three times, leaving a green solid. The solid was recrystallized by cooling a saturated solution of the product in THF to -35° C overnight, yielding green crystals of **3** (0.354 g, 47 % yield). ^1H NMR (400 MHz, C_6D_6): 6.08 (br), 5.55 (br), 3.56 (w), 1.60 (br), 1.38 (br), 1.24 (br), 0.8619 (br). IR (KBr): 2964 (s), 2933 (s), 2869 (w), 2555 (w), 2493 (w), 1534 (w), 1464 (w), 1361 (s), 1302 (w), 1182 (s), 1060 (w), 791 (w), 645 (w) cm^{-1}. μ_{eff} (293 K) = 4.0(1) μ_B. M.p.: 237 – 238 °C. MS (LIFDI, toluene): *m/z* 1146.7609 [M^+]; calcd: 1146.7565. Anal. Calcd. for $C_{60}H_{104}N_{14}B_2Cr_2$: C, 62.82; H, 9.14: N, 17.09. Found: C, 60.52; H, 9.35; N, 13.59 and C, 60.15; H, 8.63; N, 13.67. Note: this complex is air sensitive and highly reactive. Results match complex with formula: $C_{30}H_{52}N_6BCrO_2$ which is the product which forms when $[Tp^{tBu,iPr}Cr]_2(\mu$-$N_2)$ is exposed to dioxygen. Anal. Calcd. for $C_{30}H_{52}N_6BCrO_2$: C, 60.90; H, 8.86: N, 13.59.

Preparation of $[Tp^{iPr2}Cr]_2(\mu$-$N_2)$ (4)

$[Tp^{iPr2}Cr]_2(\mu$-$Cl)_2$ (1.00 g, 0.903 mmol)[42] and 2.3 equivalents of KC_8 (0.281 g, 2.079 mmol) were added to a glass ampoule and placed under vacuum. In another glass ampoule, 100 mL of a Et_2O/THF mixture (v : v = 4 : 1) was degassed. This solvent mixture was vacuum-transferred to the ampoule containing both $[Tp^{iPr2}Cr]_2(\mu$-$Cl)_2$ and KC_8. Into this same ampoule, an atmosphere of high purity N_2 was introduced. The solution turned from blue to purple over the course of 5 minutes. The solution was allowed to stir for an additional 6 hours to ensure complete conversion of $[Tp^{iPr2}Cr]_2(\mu$-$Cl)_2$. The solvents were removed under vacuum. Toluene was added to the purple residue and the solution was warmed to a gentle reflux. Graphite and KCl salt were then removed via filtration. The toluene solvent was removed under vacuum to yield a purple residue. This was recrystallized by cooling a saturated solution of the product in pentanes to -35 °C overnight as purple crystals (0.605 g, 63% yield). ^1H NMR (400 MHz, C_6D_6): 6.30 (6H), 6.20 (3H), 1.90 (18H), 1.49 (18H) ppm. IR (KBr): 2964 (s), 2929 (s),

2868 (w), 2537 (w), 1535 (s), 1471 (s), 1392 (s), 1301 (w), 1175 (s) 1052 (s), 788 (s), 659 (w) cm^{-1}. μ_{eff} (293 K) = 4.0(1) μ_B. M.p.: 219 – 220 °C. MS (LIFDI, toluene): *m/z* 1062.6652 [M^+]; calcd: 1062.6626. Anal. Calcd. for $C_{54}H_{92}N_{14}B_2Cr_2$: C, 61.01; H, 8.72: N, 18.45. Found: C, 59.05; H, 8.28; N, 15.15. Note: this complex is air sensitive and highly reactive. Results match complex with formula $C_{27}H_{46}N_6BCrO_2$ which is the product formed when $[Tp^{i\text{-}Pr2}Cr]_2(\mu\text{-}N_2)$ is exposed to dioxygen. Anal. Calcd. for $C_{27}H_{46}N_6BCrO_2$: C, 59.02; H, 8.44: N, 15.29.

Preparation of $Tp^{iPr2}Cr(CO)_3$ (5)

$[Tp^{iPr2}Cr]_2(\mu\text{-}N_2)$ (0.300 g, 0.281 mmol) was dissolved in 50 mL of toluene and added to a glass ampoule under a N_2 atmosphere. The ampoule was taken out of the inert atmosphere glove-box and the purple solution was then freeze-pump-thawed three times removing the N_2 atmosphere. To this evacuated vessel, 1 atm of CO was added, turning the solution from purple to yellow immediately. The solution was allowed to stir for an additional hour. The CO atmosphere was removed *via* 3 cycles of freeze-pump-thaw. The ampoule was brought back into the inert atmosphere glove-box. The solvent was removed under vacuum to yield a yellow residue. This was recrystallized by cooling a saturated solution of the product in THF to -35°C overnight to yield yellow crystals o **5** (0.233 g, 67 % yield). ^1H NMR (400 MHz, THF-d_8): 14.9 (br, 6H), 2.7 (br, 18H), 2.1 (br, 18H), 0.8 (br, 3H) ppm. IR (KBr): 2968 (s), 2925 (s), 2863 (w), 2548 (w), 2009 (s), 1878 (s), 1842 (w), 1534 (s), 1476 (s), 1391 (s), 1391 (w), 1301 (s), 1177 (s) 1051 (s), 791 (s), 658 (w) cm^{-1}. μ_{eff} (293K) = 1.8(1) μ_B. M.p.: 263 - 265°C. MS (LIFDI, toluene): *m/z* 601.3149 [M^+]; calcd: 601.3130. Anal. Calcd. for $C_{30}H_{46}N_6BCrO_3$: C, 59.90; H, 7.71: N, 13.18. Found: C, 60.38; H, 7.82; N, 13.18.

Preparation of $Tp^{tBu,Me}Cr(\eta^2\text{-}C_2(SiMe_3)_2)$ (6)

$Tp^{tBu,Me}Cr(THF)Cl$ (0.600 g, 1.175 mmol), 1.1 equivalents of *bis*-(trimethylsilyl)acetylene (0.219 g, 1.290 mmol), and 1.2 equivalents of KC_8 (0.190 g, 1.410 mmol) were added to a glass ampoule under an N_2 atmosphere in an inert-atmosphere glove-box. The ampoule was then evacuated and to this vessel 50 mL of a degassed Et_2O/THF mixture (v : v = 4 : 1) was added *via* vacuum transfer. The solution

was allowed to warm to room temperature producing a brown colored solution. The solution was allowed to stir for 6 hours. The solvents were then removed to yield a brown residue. The solid was redissolved into pentanes and the solution was filtered to remove graphite and KCl. The product was recrystallized by cooling a saturated pentanes solution of the product to -35° C overnight, to yield brown crystals of **6** (0.566 g, 75 % yield). ^{1}H NMR (400 MHz, C_6D_6): 34.8 (9H), 12.2 (3H), 2.1 (27H), -7.5 (18H) ppm. IR (KBr): 2957 (s), 2929 (s), 2866 (w), 2534 (w), 1707 (w), 1539 (s), 1466 (w), 1428 (s), 1361 (s), 1241 (s), 1161 (s), 1060 (s), 852 (s), 832 (s), 784 (s), 764 (s), 644 (w) cm^{-1}. μ_{eff} (293 K) = 3.6(1) μ_B. M.p.: 190 - 192°C. MS (LIFDI, toluene): *m/z* 645.3783 [M^+]; calcd: 645.3760. Anal. Calcd. for $C_{32}H_{58}N_6BCrSi2$: C, 59.51; H, 9.05: N, 13.01. Found: C, 59.31; H, 8.97; N, 13.23.

Preparation of [Tp^{tBu,Me}Cr]$_2$(η^1-CO)$_2$(μ-CO)(Et_2O) (7)

Tp^{tBu,Me}Cr(η^2 - $C_2(SiMe_3)_2$) (0.500 g, 0.771 mmoles) was dissolved in Et_2O and added to a glass ampoule under an N_2 atmosphere. The solution was degassed through three cycles of freeze-pump-thaw. To the evacuated vessel, 1 atm of CO was added, immediately turning the color of the solution from brown to yellow. The solution was allowed to stir for 6 hours. The CO gas and the solvent were then removed to yield a yellow solid. This was recrystallized by cooling a saturated Et_2O solution to -35° C overnight, yielding yellow crystals of **7** (0.519 g, 65 % yield). ^{1}H NMR (400 MHz, C_6D_6): 6.0 (br), 5.8 (br), 5.2 (br), 2.8 (br), 1.8 (br), 1.4 (br), 1.2 (br), 0.3 (br) ppm. IR (KBr): 2961 (s), 2923 (s), 2867 (w), 2547 (w), 2033 (s), 1955 (s), 1865 (s), 1539 (s), 1480 (w), 1422 (s), 13.42 (s), 1182 (s), 845 (s), 832 (s), 792 (w), 647 (w) cm^{-1}. μ_{eff}(293 K) = 4.9(1) μ_B. M.p.: 188 - 190°C. MS (LIFDI, toluene): *m/z* 1034.5490 [M^+-Et_2O]; calcd: 1034.5473. Anal. Calcd. for $C_{55}H_{90}N_{12}B_2Cr_2O_4$: C, 59.57; H, 8.18: N, 15.16. Found: C, 59.19; H, 8.13; N, 14.67.

Preparation of [Tp^{tBu,Me}Cr]$_2$(μ_2-η^2:η^2-C_2H_4) (8)

Tp^{tBu,Me}Cr(η^2-$C_2(SiMe_3)_2$) (0.300 g, 0.465 mmol) was dissolved into 50 mL of pentanes and added to a glass ampoule under an N_2 atmosphere. The solution was degassed through three cycles of freeze-pump-thaw. To the evacuated vessel, 1 atm of

ethylene was added, which turned the solution from brown to green over the course of 1 hour. The solution was allowed to stir for an additional 6 hours. The ethylene gas and solvent were removed to yield a green solid. The green solid was recrystallized by cooling a saturated pentanes solution of the product to -35° C overnight, to yield trichroic(!) (green-purple-blue) crystals of **8** (0.152 g, 67 % yield). ^{1}H NMR (400 MHz, C_6D_6): 14.4 (9H), 3.2 (3H), 0.8 (27H) ppm. IR (KBr): 2958 (s), 2928 (s), 2863 (w), 2476 (w), 1540 (s), 1461 (w), 1430 (s), 1358 (s), 1193 (s), 1070 (s), 1058 (s), 1027 (w), 781 (s), 631 (w) cm^{-1}. μ_{eff} (293 K) = 3.7(3) μ_B. M.p.: 198 - 200 °C. MS (LIFDI, toluene): *m/z* 984.6017; LIFDI-MS analysis was consistent with $[Tp^{tBu,Me}Cr]_2(\mu\text{-}OH)_2$ on three different attempts; M^+ calcd: 984.56801 . **8** is too sensitive and reactive (with H_2O?) to be characterized by LIFDI-MS. Anal. Calcd. for $C_{50}H_{84}N_{12}B_2Cr_2$: C, 61.35; H, 8.65: N, 17.17. Found: C, 57.77; H, 8.15; N, 16.30. The elemental analysis is also more consistent with $[Tp^{tBu,Me}Cr]_2(\mu\text{-}OH)_2$. Anal. Calcd. for $C_{48}H_{82}N_{12}B_2Cr_2O_2$: C, 58.54; H, 8.39; N, 17.07.

Preparation of $Tp^{tBu,Me}Cr(\eta^2\text{-}C_2(CH_3)_2)$ (9)

$Tp^{tBu,Me}Cr(THF)Cl$ (0.500g, 0.858mmol) and KC_8 (0.138g, 1.029mmol) were added to a glass ampoule under an N_2 atmosphere in an inert-atmosphere glove-box. The ampoule was then evacuated under vacuum and to this vessel 50 mL of degassed Et_2O – THF mixture (v : v = 4 : 1) was *via* condensation under vacuum. The solution was allowed to warm to room temperature yielding a tan colored solution. 1 atm of argon gas was introduced to this vessel. One equivalent of degassed 2-butyne (0.046g, 0.858mmol) was then added to the vessel turning the tan colored solution yellow. The solution was allowed to stir for 6 hours. The solvents were then removed to yield a yellow solid. The yellow residue was redissolved into pentanes. The solution was filtered to remove graphite and KCl salt. The solvent was removed to yield a yellow solid. $Tp^{tBu,Me}Cr(\eta^2\text{-}C_2(CH_3)_2)$ was recrystallized by cooling a saturated pentanes solution of the product to -35° C overnight to give orange crystals (0.275g, 52 % yield). ^{1}H NMR (400 MHz, C_6D_6): 45.4 (9H), 8.0 – 2.0 (30H), and -5.5 (6H) ppm. IR (KBr): 2960 (s), 2921 (s), 2863 (w), 2548 (w), 2478 (w), 1541 (s), 1477 (w), 1422 (s), 1361 (s), 1361 (s), 1198 (s), 1063 (s), 972 (s), 945 (s), 789 (s), 647 (w) cm^{-1}. μ_{eff} (293K) = 3.7(1)μ_B. M.p.: 185 -

188 °C. MS (LIFDI, toluene): *m/z* 529.3279 [M^+]; calcd: 529.3282. Anal. Calcd. for $C_{28}H_{46}N_6BCr$: C, 63.51; H, 8.76: N, 15.87. Found: C, 63.29; H, 8.63; N, 15.81.

Preparation of $Tp^{tBu,Me}Cr(\eta^2\text{-}C_2(C_6H_5)_2)$ (10)

$Tp^{tBu,Me}Cr(THF)Cl$ (0.500g, 0.858mmol), one equivalent of diphenylacetylene (0.152g, 0.858mmol), and 1.2 equivalents of KC_8 (0.138g, 1.029mmol) were added to a glass ampoule under an N_2 atmosphere in an inert-atmosphere glove-box. The ampoule was evacuated and to this vessel 50 mL of degassed Et_2O – THF mixture (v : v = 4 : 1) was added *via* vacuum transfer. The solution was allowed to warm to room temperature yielding a yellow colored solution. The solution was allowed to stir for 6 hours. The solvents were removed to yield a dark tan solid. The residue was redissolved into pentanes and the solution was filtered to remove graphite and KCl salt. The solvent was removed to produce a yellow solid. $Tp^{tBu,Me}Cr(\eta^2\text{-}C_2(C_6H_5)_2)$ was recrystallized by cooling a saturated pentanes solution to -35° C overnight to yield yellow crystals (0.358g, 56% yield). ^{1}H NMR (400 MHz, C_6D_6): 45.3 (3H), 19.2 (5H), 6.2 to -5.9 (27H), -2.1 (5H), and -7.2 (9H) ppm. IR (KBr): 2960 (s), 2930 (s), 2865 (w), 2547 (w), 2489 (w), 1601 (w), 1540 (s), 1422 (w), 1260 (s), 1195 (s), 1069 (s), 1026 (s), 794 (s), 756 (s), 690 (s), 647 (w) cm^{-1}. μ_{eff}(293K) = 3.7(1) μ_B. M.p.: 158 - 160 °C. MS (LIFDI, toluene): *m/z* 653.3603 [M^+]; calcd: 653.3595. Anal. Calcd. for $C_{38}H_{50}N_6BCr$: C, 69.82; H, 7.71: N, 12.86. Found: C, 69.87; H, 7.66; N, 12.57.

X-ray diffraction studies. X-ray structural analysis for **1 – 10**. Crystals were mounted using viscous oil onto a plastic mesh and cooled to the data collection temperature. Data were collected on a Bruker-AXS CCD diffractometer. Unit cell parameters were obtained from 36 data frames, 0.5° ω, from three different sections of the Ewald sphere. No symmetry higher than triclinic was observed for **1, 3, 6, 7,** and **8**. The equivalent reflections and systematic absences in the diffraction data are consistent with *P3* and *P-3* for **5**, and, uniquely, for $P2_1/n$ for **2**, **9**, and **10**. Solution in the centrosymmetric space group options yielded chemically reasonable and computationally stable results of refinement for **5** and **t**he triclinic cases. The data-sets were treated with multi-scan

absorption corrections (Apex2 software suite, Madison, WI, 2005). The structures were solved using direct methods and refined with full-matrix, least-squares procedures on F^2.[6]

The compound molecule is located at a threefold axis in **5** and at inversion centers in **1, 3,** and **4**. One water and one diethyl ether per compound molecule in **5** were treated as diffused contributions. (Squeeze, Platon).[7] Two molecules of cocrystallized THF solvent molecules in **1**, each symmetry unique isopropyl group in **3**, a pyrazole group and a THF solvent molecule in **4,** two t-butyl groups in **6** and a t-butyl group in **7** were located disordered in two positions and either treated as an idealized rigid group (for THF molecules) or treated with noncrystallographic, chemical symmetry and anisotropic rigid bond restraints.

Each unique disordered isopropyl moiety in **3** yielded refined site occupancy ratios of 62/38, 67/33 and 88/12. Two half-occupied molecules of diethylether were located in the asymmetric unit of **3** and treated with anisotropic rigid bond restraints.

One of the unique pyrazole groups in **4** was located disordered with a refined site occupancy distribution of 63/37. A disordered THF of solvation was located in **4**, treated with a refined site occupancy of 60/40. A global 3-dimensional anisotropic rigid bond restraint was applied to **4**.

One of the two compound molecules located in the asymmetric unit of **6** displays two disordered t-butyl groups with refined site occupancies of 66/34 and 71/29.

A t-butyl group in **7** was found disordered a refined site occupancy distribution of 77/23. A diethyl ether molecule of solvation was located in the asymmetric unit of **7** and treated as a flat molecule with similar 1,2 and 1,3 distances.

A half-molecule of noncoordinated diphenyl acetylene and a THF molecule were found cocrystallized in the asymmetric unit of **10**. The THF solvent molecule in **10** was treated as an idealized rigid group with rigid bond anisotropic restraints.

The hydrogen atoms of the bridging ethylene in **8** were located from the difference map and restrained as a flat molecule with similar C-H bond and geminal H…H atom distances. The borohydride H-atom was located in **1**, **2**, **5**, **6**, **7**, **8**, and **9** and constrained to 1.2 of the equivalent isotropic parameter of the attached boron atom.

Although all methyl H-atoms were allowed to rotate along the C-C bond, an anomalous short H…H distance was observed in **7** involving one of the disordered t-butyl groups which caused a level B alert in the checkCIF report (http://checkcif.iucr.org). All non-hydrogen atoms were refined with anisotropic displacement parameters. All other remaining hydrogen atoms were treated as idealized contributions with geometrically calculated positions and with U_{iso} equal to 1.2, or 1.5 for methyl, U_{eq} of the attached atom. Atomic scattering factors are contained in various versions of the SHELXTL program library.[6] The CIF has been deposited under CCDC 1058333-1058342.

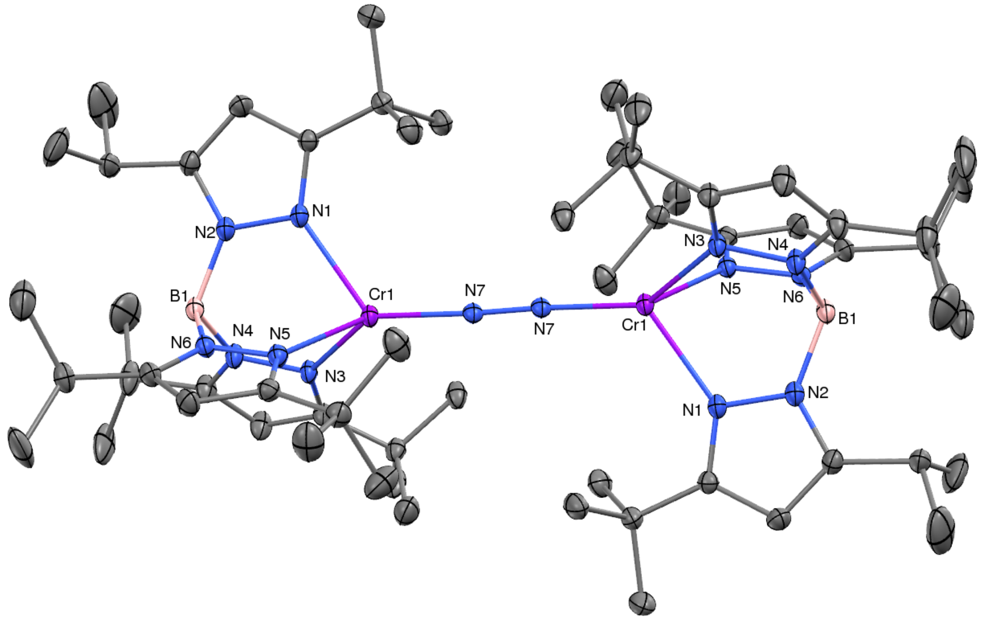

Figure S1. The molecular structure of $[Tp^{tBu,iPr}Cr]_2(\mu\text{-}N_2)$ (**3**, 30% probability level). Selected interatomic distances (Å) and angles (º): N7-N7A, 1.209(3); Cr-N7, 1.8395(16), Cr-N1, 2.1922(17); Cr-N3, 2.2020(17); Cr-N5, 2. 1795(17); N_{Tp}-Cr-$N_{Tp,avg}$, 87.7; N_{Tp}-Cr-$N7_{avg}$, 127.3.

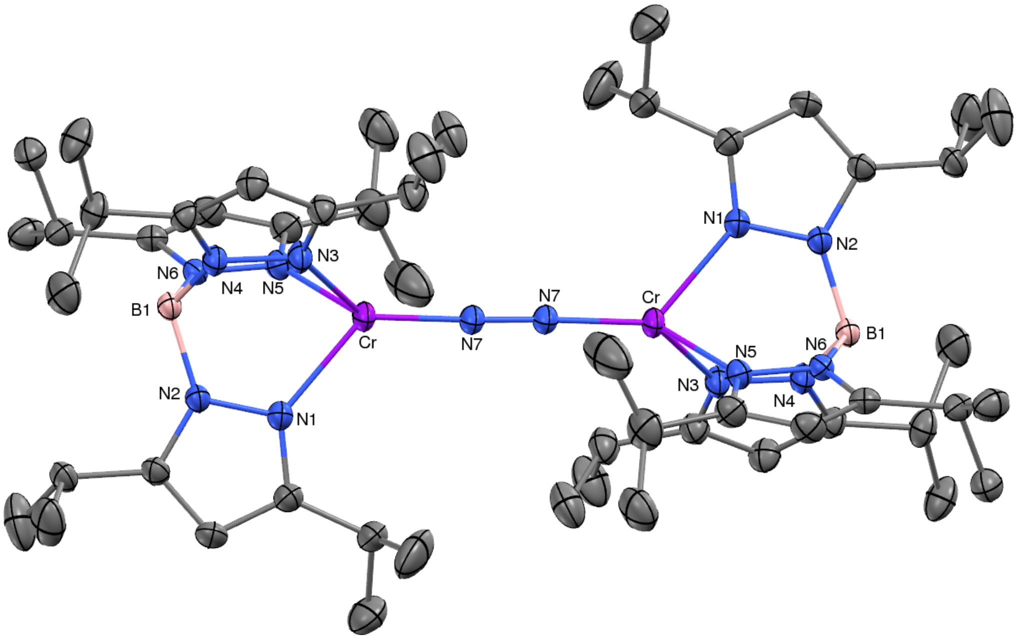

Figure S2. The molecular structure of $[Tp^{iPr,iPr}Cr]_2(\mu\text{-}N_2)$ (**4**, 30% probability level). Selected interatomic distances (Å) and angles (º): N7-N7A, 1.214(4); Cr-N7, 1.773(2), Cr-N1, 2.103(2); Cr-N3, 2.110(2); Cr-N5, 2. 068(17); N_{Tp}-Cr-$N_{Tp,avg}$, 87.3; N_{Tp}-Cr-$N7_{avg}$, 127.1.

a)

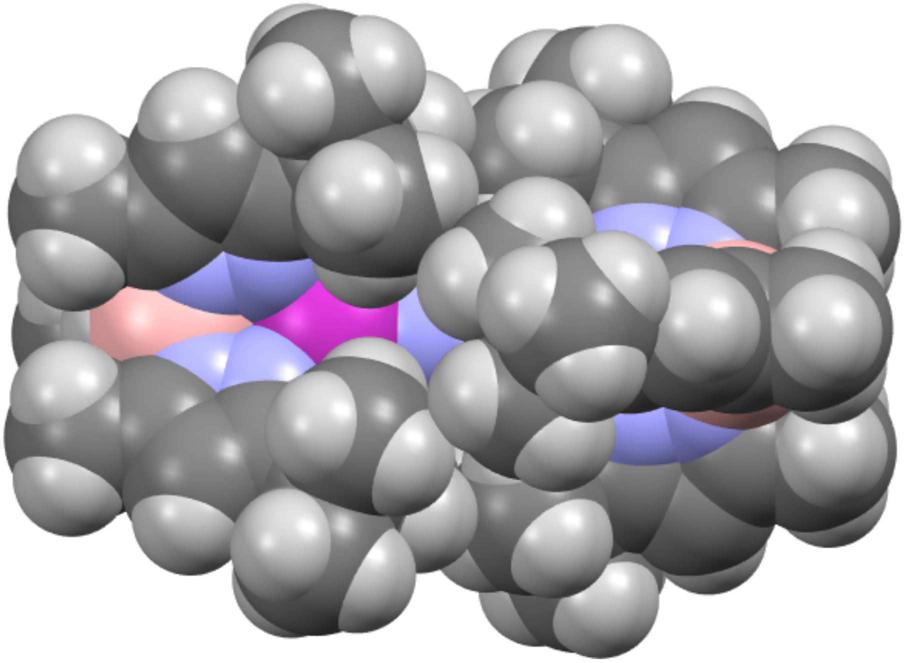

b)

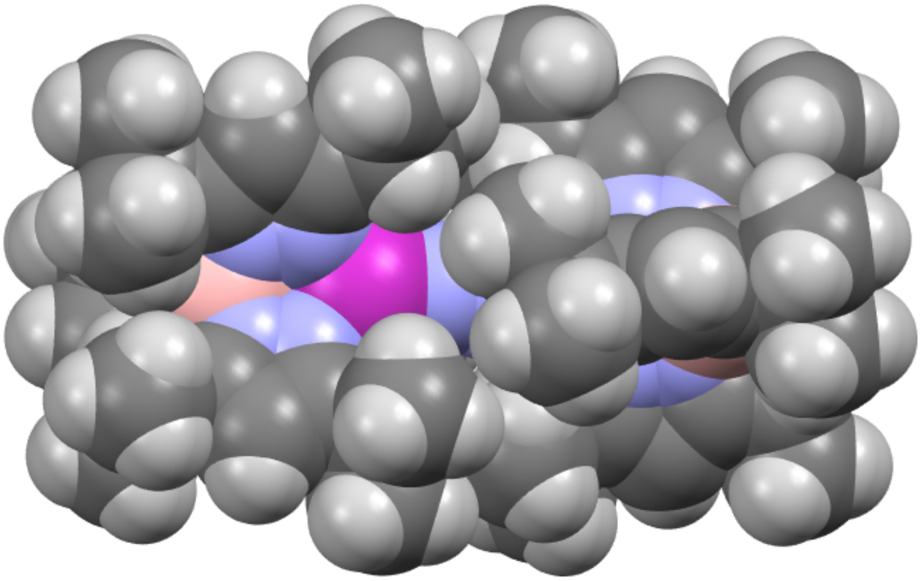

Figure S3. Space filling representations of a) $[Tp^{tBu,Me}Cr]_2(\mu\text{-}\eta^1\text{:}\eta^1\text{-}N_2)$ (**1**) and b) $[Tp^{iPr,iPr}Cr]_2(\mu\text{-}N_2)$ (**4**). View is down the trajectory of a ligand approaching the left chromium (colored magenta)

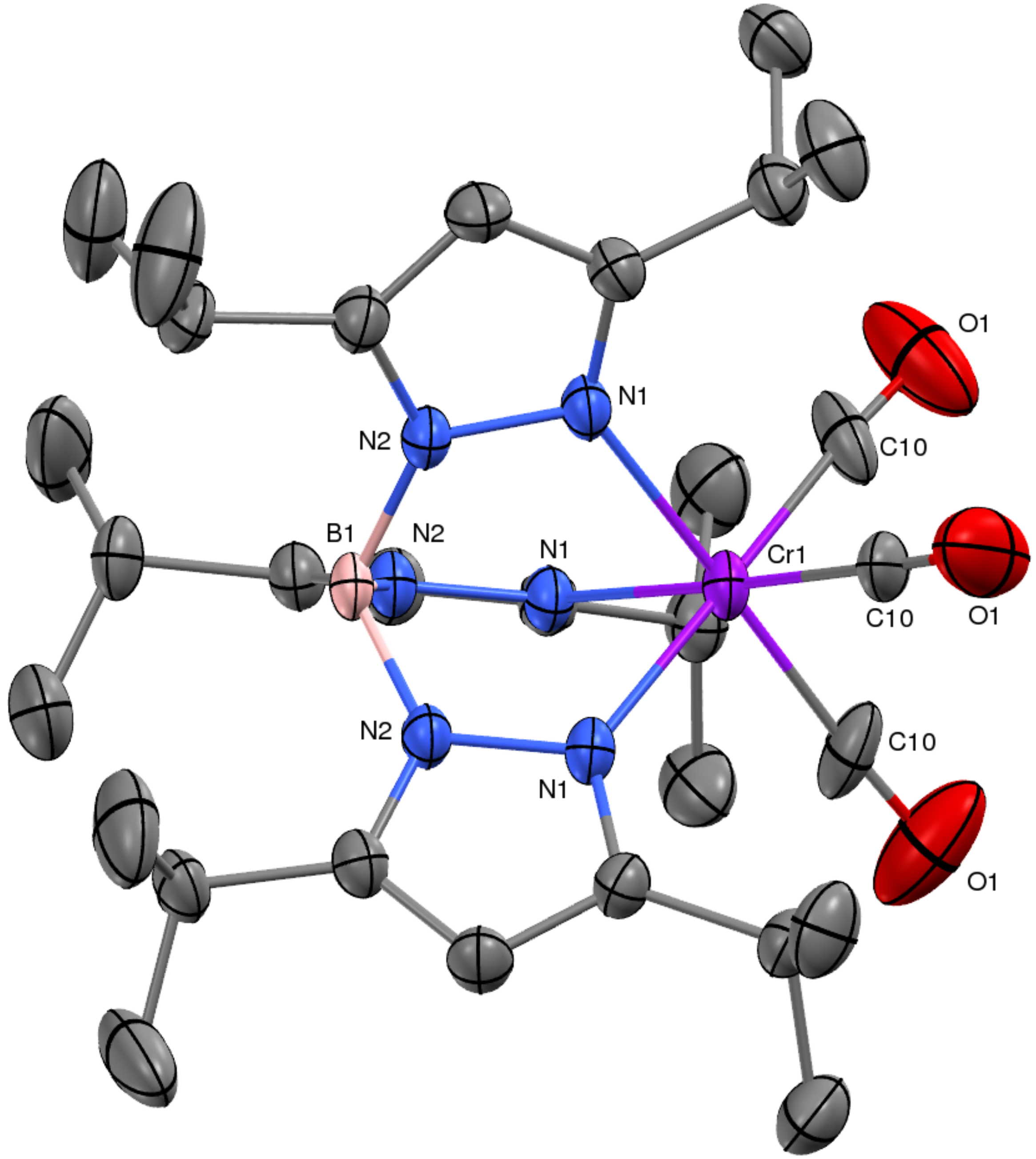

Figure S4. The molecular structure of $Tp^{iPr,iPr}Cr(CO)_3$ (**5**, 30% probability level). Selected interatomic distances (Å) and angles (°): Cr – C10, 1.895(5); C10-O1, 1.104(5), Cr-N1, 2.100(3); C10-Cr-C10a, 88.0(2); N1-Cr-N1a, 86.70(11).

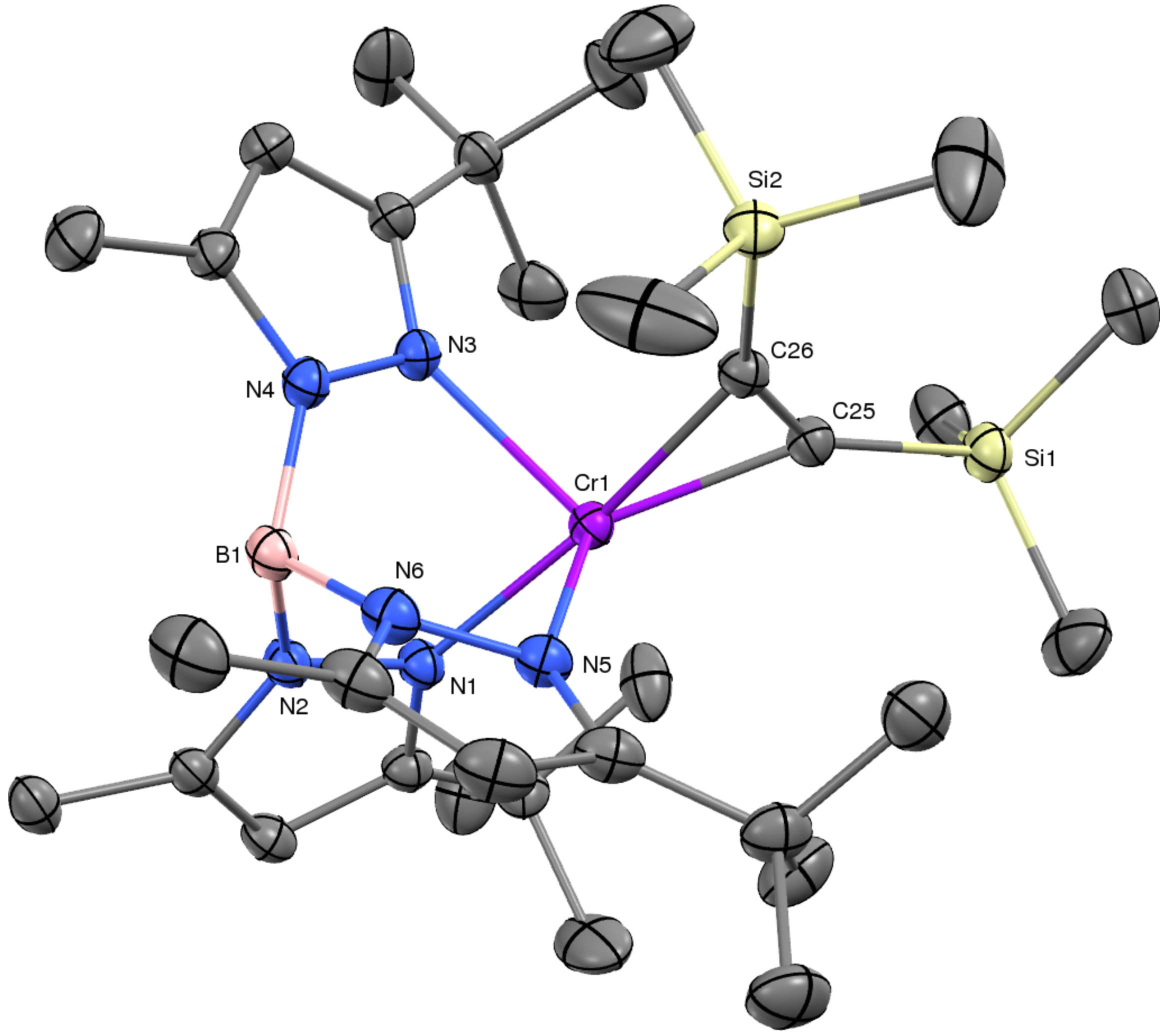

Figure S5. The molecular structure of $Tp^{tBu,Me}Cr(\eta^2\text{-}C_2(SiMe_3)_2)$ (**6**, 30% probability level). Selected interatomic distances (Å) and angles (°): Cr-C25, 2.0480(19); Cr-C26, 2.0835(18); C25-C26, 1.288(3); Cr-N1, 2.1015(15); Cr-N3, 2.1614(16); Cr-N5, 2. 1504(16); N_{Tp}-Cr-$N_{Tp,avg}$, 87.7; N1-Cr-C25/$C26_{centroid}$, 172.5; α (angle of deviation of alkyne centroid from B-Cr axis) = 49.3°.

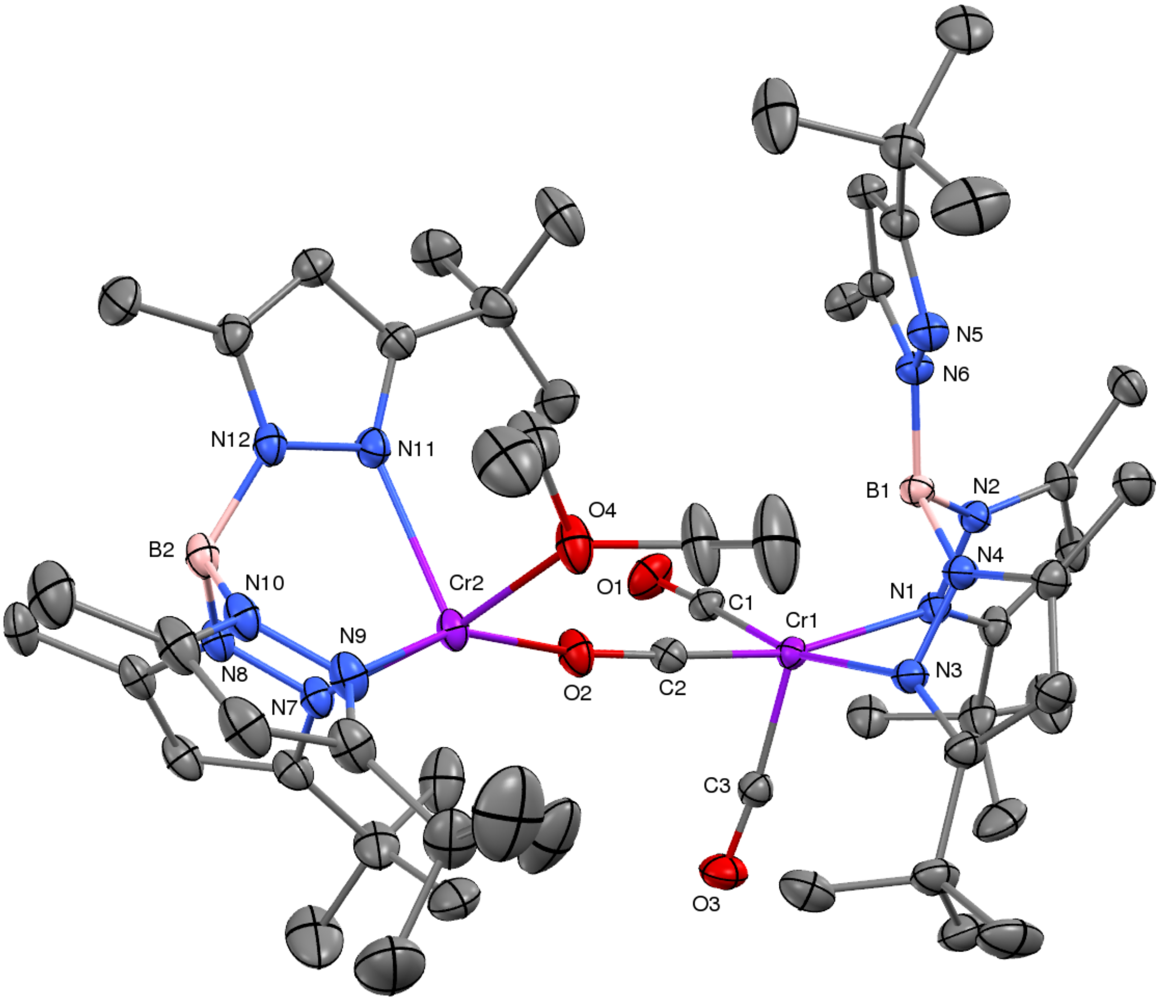

Figure S6. The molecular structure of κ^2-$Tp^{tBu,Me}Cr(CO)_2(\mu$-η^1:η^1-CO)$(Et_2O)CrTp^{tBu,Me}$ (**7**, 30% probability level). Selected interatomic distances (Å) and angles (º): Cr1 – C2, 1.757(2); C2-O2, 1.214(3); Cr-C1, 1.833(2); C1-O1, 1.159(3); Cr1-C3, 1.832(2); C2-O3, 1. 163(3); Cr1-N1, 2.1944(18); Cr1-N3, 2.1813(18); Cr2-O2, 2.0325(16), Cr2-O4, 2.1333(17); 2.100(3); Cr2-O2-C2, 157.23(16); Cr1-C2-O2, 173.34(19).

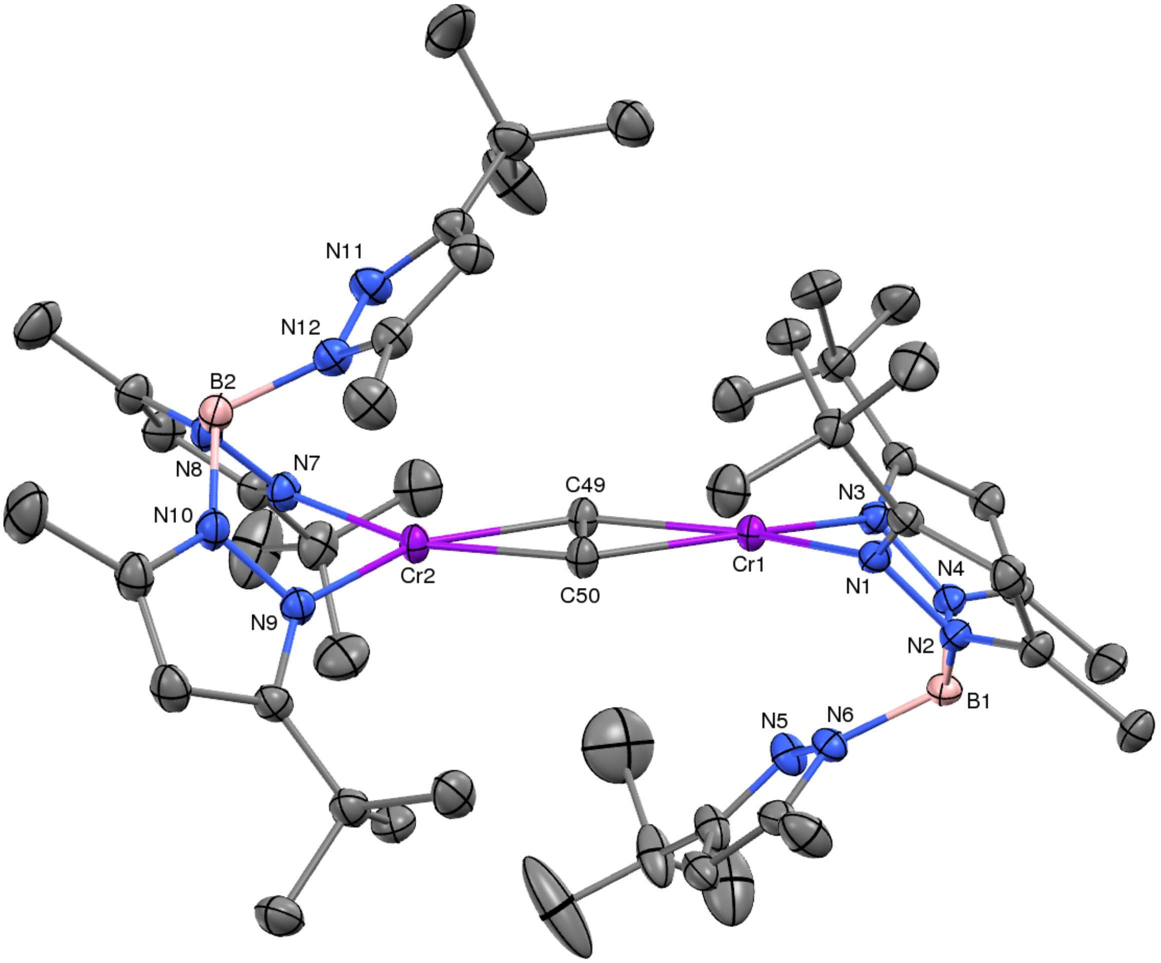

Figure S7. The molecular structure of [κ^2-Tp^{tBu,Me}Cr]$_2$(μ-η^2:η^2-C_2H_4) (**8**, 30% probability level). Selected interatomic distances (Å): Cr1-C49, 2.178(3); Cr1-C50, 2.170(3); Cr-C49, 2.191(3); Cr2-C50, 2.155(3), C49-C50, 1.487(4); Cr1-N1, 2.107(2); Cr1-N3, 2.098(2); Cr2-N7, 2. 118(2); Cr2-N9, 2.101(2).

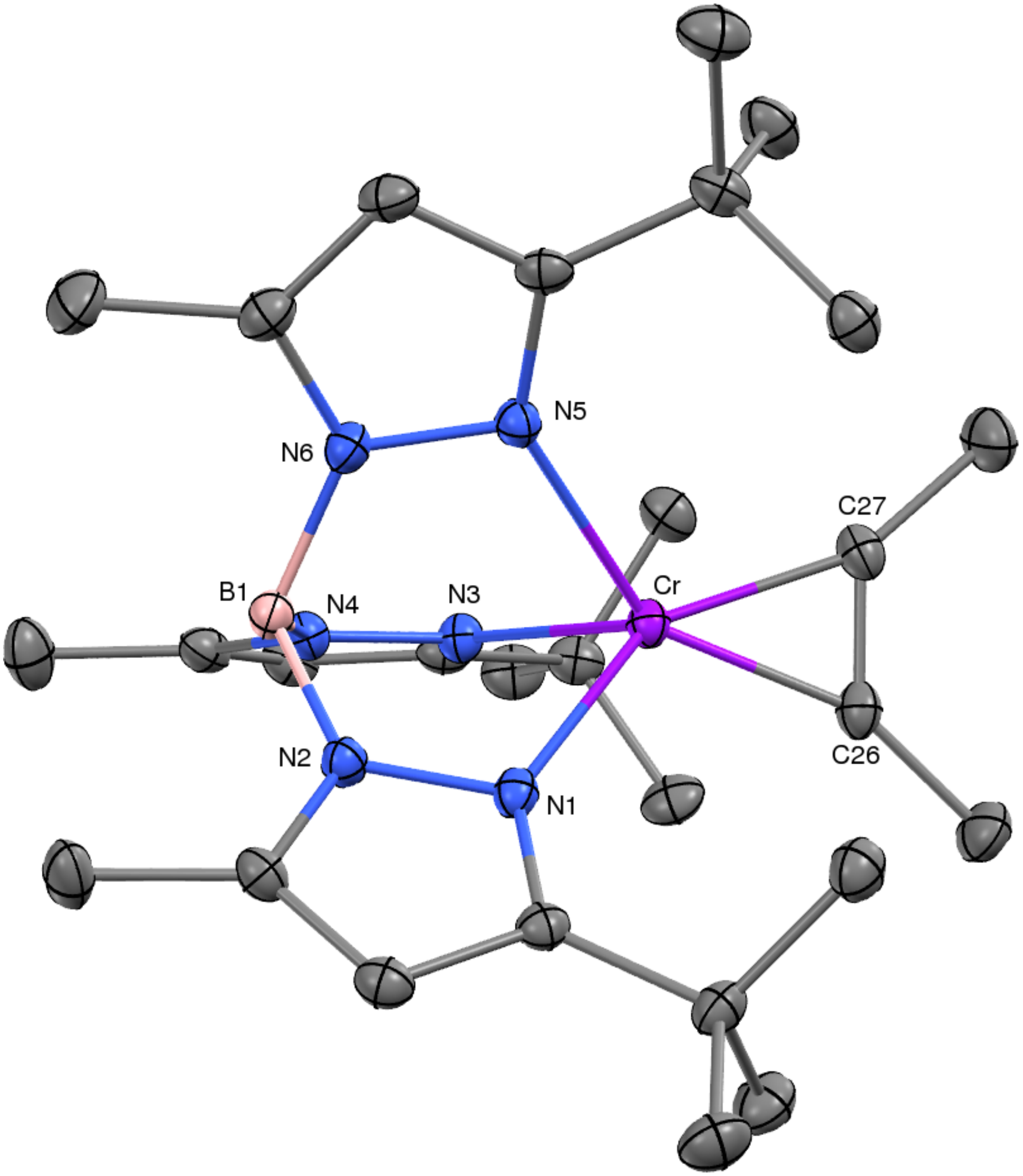

Figure S8. The molecular structure of $Tp^{tBu,Me}Cr(\eta^2\text{-}C_2Me_2)$ (**9**, 30% probability level). Selected interatomic distances (Å) and angles (º): Cr-C26, 1.955(4); Cr-C27, 1.956(4); C26-C27, 1.308(5); Cr-N1, 2.204(3); Cr-N3, 2.104(3); Cr-N5, 2. 201(3); N_{Tp}-Cr-$N_{Tp,avg}$, 88.7; α (angle of deviation of alkyne centroid from B-Cr axis) = 5.2º.

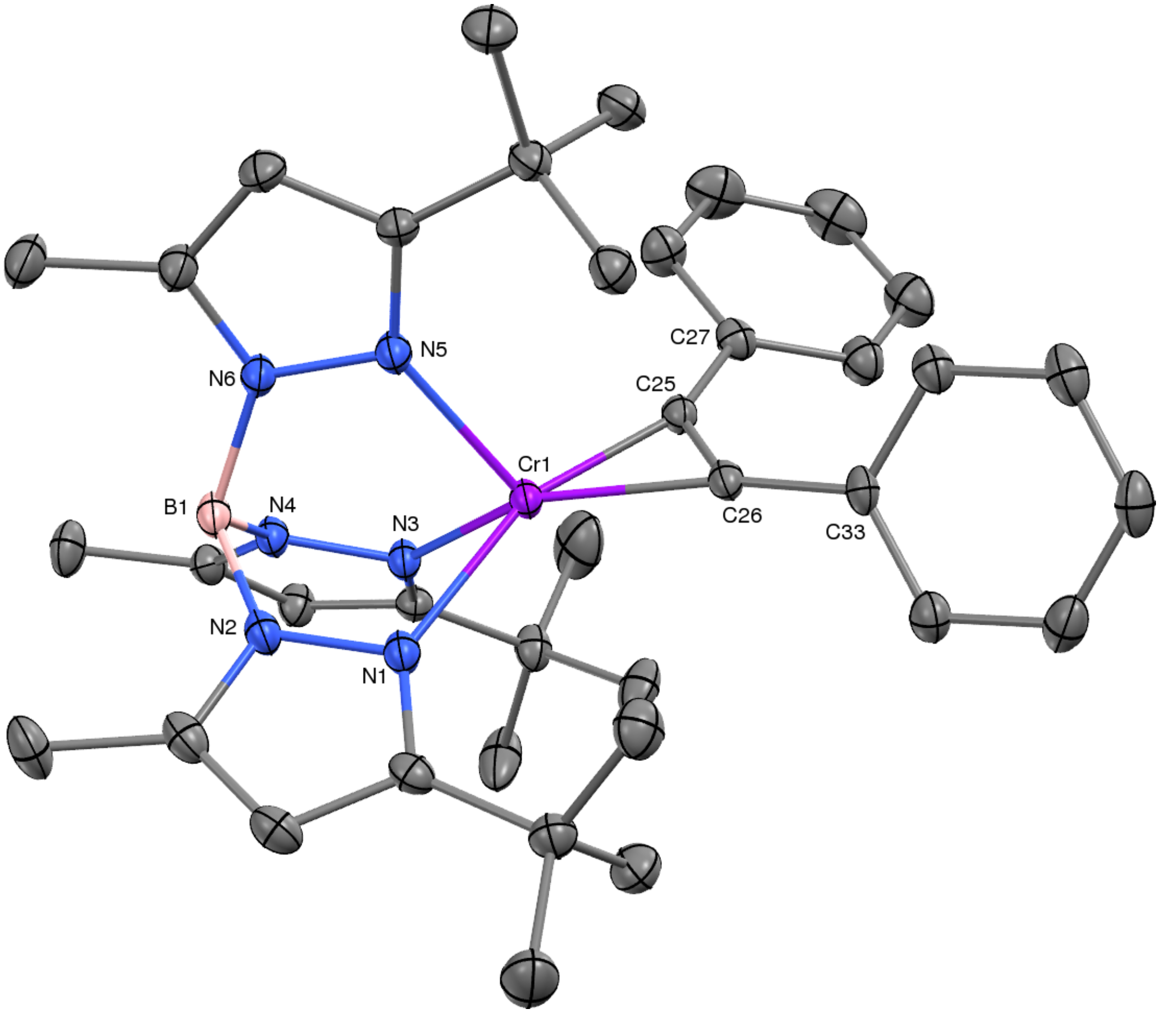

Figure S9. The molecular structure of $Tp^{tBu,Me}Cr(\eta^2\text{-}C_2Ph_2)$ (**10**, 30% probability level). Selected interatomic distances (Å) and angles (°): Cr-C25, 1.941(2); Cr-C26, 1.989(2); C25-C26, 1.322(4); Cr-N1, 2.201(2); Cr-N3, 2.184(2); Cr-N5, 2. 087(2); N_{Tp}-Cr-$N_{Tp,avg}$, 89.0; α (angle of deviation of alkyne centroid from B-Cr axis) = 16.0°.

References:

(1) Kersten, J. L.; Kucharczyk, R. R.; Yap, G. P. A.; Rheingold, A. L.; Theopold, K. H. *Chem. Eur. J.* **1997**, *3*, 1668-1674.
(2) Sugawara, K.; Hikichi, S.; Akita, M. *J. Chem. Soc., Dalton Trans.* **2002**, 4514-4524.
(3) Trofimenko, S. *Chem.Rev.* **1993**, *93*, 943-980.
(4) *Handbook of Preparative Inorganic Chemistry*; 2nd ed.; Brauer, G., Ed.; Academic Press: New York, 1969; Vol. 1.
(5) Savoia, D.; Trombini, C.; Umanironchi, A. *Pure Appl. Chem.* **1985**, *57*, 1887-1896.
(6) Sheldrick, G. M. *Acta Crystallographica Section A* **2008**, *64*, 112-122.
(7) Spek, A. L. *J. Appl. Crystallogr.* **2003**, *36*, 7-13.
